# Improving the intrinsic activity of electrocatalysts for sustainable energy conversion: where are we and where can we go?

**DOI:** 10.1039/d1sc04775b

**Published:** 2021-11-23

**Authors:** Nitish Govindarajan, Georg Kastlunger, Hendrik H. Heenen, Karen Chan

**Affiliations:** Catalysis Theory Center, Department of Physics, Technical University of Denmark (DTU) Fysikvej 311 2800 Kgs. Lyngby Denmark kchan@fysik.dtu.dk; Fritz-Haber-Institut der Max-Planck-Gesellschaft Faradayweg 4–6 D-14195 Berlin Germany

## Abstract

As we are in the midst of a climate crisis, there is an urgent need to transition to the sustainable production of fuels and chemicals. A promising strategy towards this transition is to use renewable energy for the electrochemical conversion of abundant molecules present in the earth's atmosphere such as H_2_O, O_2_, N_2_ and CO_2_, to synthetic fuels and chemicals. A cornerstone to this strategy is the development of earth abundant electrocatalysts with high intrinsic activity towards the desired products. In this perspective, we discuss the importance and challenges involved in the estimation of intrinsic activity both from the experimental and theoretical front. Through a thorough analysis of published data, we find that only modest improvements in intrinsic activity of electrocatalysts have been achieved in the past two decades which necessitates the need for a paradigm shift in electrocatalyst design. To this end, we highlight opportunities offered by tuning three components of the electrochemical environment: cations, buffering anions and the electrolyte pH. These components can significantly alter catalytic activity as demonstrated using several examples, and bring us a step closer towards complete system level optimization of electrochemical routes to sustainable energy conversion.

## Abstract 2

We evaluate the improvements over the past two decades in intrinsic activity of electrocatalysts for sustainable energy conversion, and highlight opportunities from tuning the electrolyte.

## Graphical abstract

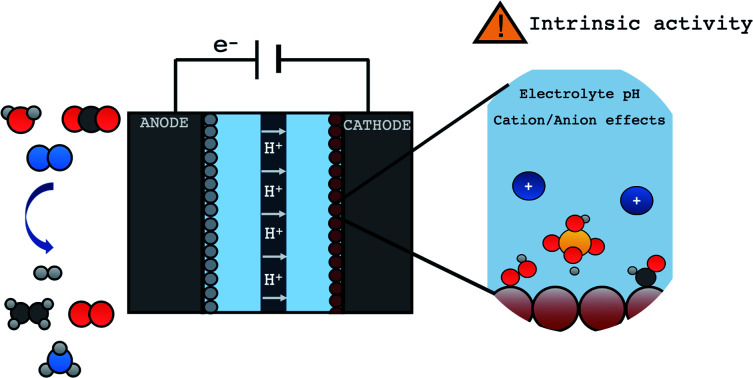

## Introduction

With the continuous growth in global population and projected increase in the global energy consumption by 50% within the next three decades, we are at a defining moment of the climate change crisis.^2^ A vast majority (>80%) of the global energy demand is currently derived from fossil fuels, which are the primary cause of the increase in anthropogenic CO_2_ emissions. Thus, there is an urgent need for a transition towards the sustainable production of fuels and chemicals.

A promising strategy towards the transition to a sustainable economy is the electrochemical conversion of molecules present in the earth's atmosphere such as H_2_O, O_2_ and CO_2_.^1^ Central reactions in such conversion schemes are: (1) the water splitting reaction, which consists of the oxygen evolution (OER) and hydrogen evolution (HER) to produce hydrogen, (2) oxygen reduction (ORR) to water and hydrogen oxidation (HOR), reactions at the heart of fuel cells and metal–air batteries, and (3) carbon dioxide reduction (CO_2_RR) to high value fuels and chemicals such as CO, C_1_ products (methane and methanol) and C_2_ products (ethylene, acetate and ethanol). In recent years, more complex electrosynthesis reactions involving the valorization of biomass feedstocks (*e.g.* redox reactions involving 5-hydroxymethylfurfural and glycerol) have also been explored towards the production of high value fuels and chemicals.^3,4^ Electrochemical conversion schemes offer several advantages over conventional thermal schemes including (i) operability at room temperature and pressure,^5^ (ii) a highly distributed infrastructure, (iii) the use of abundant H_2_O molecules instead of expensive H_2_ for hydrogenation reactions and (iv) the ability to achieve high selectivity towards the desired products preventing the production of wasteful/toxic by-products.

Crucial to enabling the widespread implementation of electrochemical energy conversion schemes is the development of earth-abundant and stable electrocatalysts with high intrinsic activity and selectivity towards the desired products. Two common strategies have been used by our community to improve the activity of an electrocatalytic system: (a) increasing the number of active sites through increased catalyst loading or meso/nano structuring (commonly referred to as roughening) and (b) discovery/design of new active sites with higher intrinsic activity. Challenges with the former strategy (a) are (1) the distribution of catalysts over electrodes of thicker width would lead to additional limitations in mass transport, (2) increased cost associated with increased loading of existing precious metal catalysts, and (3) increased loading can only improve geometric activity by up to three orders of magnitude.^6^ Therefore, improving the intrinsic activity of the electrocatalyst is crucial for the development of electrocatalytic systems with near ideal efficiencies.

The aim of this perspective is to highlight the following topics: (1) the importance of an accurate estimation of the active site density of electrocatalysts for evaluating the turnover frequency (TOF) – the metric for intrinsic activity, (2) the modest improvements in the intrinsic activity of electrocatalysts that have been achieved in the past two decades for the electrochemical conversion schemes, (3) the implications of the Arrhenius law on theoretical predictions of intrinsic activity and product selectivity, and (4) opportunities offered by the electrochemical environment to tune the catalytic activity and product selectivity: the role of interfacial pH, alkali cations and buffer species. The aforementioned topics have important implications for electrode design strategies aimed at improving intrinsic catalytic activity and product selectivity, and for theoretical efforts directed towards their predictions.

## We need active site estimations and fast mass transport to determine intrinsic catalytic activity

The intrinsic activity of an electrocatalyst is rigorously determined by estimating its per-site turnover frequency (TOF(*η*), s^−1^): the number of chemical conversions of the reactant molecule to the desired product per unit time on a single active site of the catalyst at a given overpotential *η*. However, per-site TOFs are seldom reported in the literature due to the challenges associated with the identification and quantification of the inherent activity of each type of active site present in an electrocatalyst. These challenges are further compounded by stability issues, parasitic/side reactions and mass transport limitations. Therefore, most studies report activity either in terms of the total electrode activity *j*(*η*) (eqn (1)) or *j*(*η*) normalized either by the loaded mass of the electrocatalyst (mass activity (MA), A mg^−1^, eqn (2)) or by the electrochemically active surface area (ECSA) (*j*_ECSA_, A cm^−2^, eqn (3)).^6^

In studies that report the total electrode activity, a commonly used metric for activity comparison is the potential required to reach a given (geometric) current density (*e.g.* 10 mA cm^−2^). While these measurements are less challenging as they do not involve active site estimations, they are not meaningful for intrinsic activity comparison. This apparent activity would reflect a convolution of intrinsic activity, catalyst loading, and catalyst roughness. In addition, this apparent activity, even if unaffected by mass transport, does not reveal any information about the nature of active sites (unless performed on defect-free single crystal electrodes), which are needed for atomistic insights on structure–property relationships. To obtain estimates of intrinsic activity, we need to determine the total number of active sites in order to obtain normalized current densities and average TOFs.

Mass normalization can be of practical relevance for expensive precious metal based catalysts like Pt for the HER/ORR or Ir/Ru based materials for the OER but is of lesser relevance for earth abundant/non-precious catalysts as the loaded mass is not critical to the overall device cost.^7^ For these nonprecious materials, it is a common strategy to estimate the active site density by measuring the ECSA. Electrochemical techniques used in ECSA estimations for metal surfaces include H adsorption/desorption, specific capacitance measurements and electrochemical adsorption of probe species. For instance, hydrogen underpotential deposition is widely used in measuring the surface area of Pt based materials.^8^ Other common techniques include underpotential deposition of Pb/Cu^9^ and CO stripping. It is important to note here that there can be errors associated with these estimations as the catalyst structure and/or availability of certain active sites can differ from typical electrochemical measurement conditions and the reaction conditions. Overall, while it is preferable to estimate the per-site TOF of the catalyst, mass and ECSA normalized activity are approximations that provide first indications of the intrinsic activity of an electrocatalyst.1
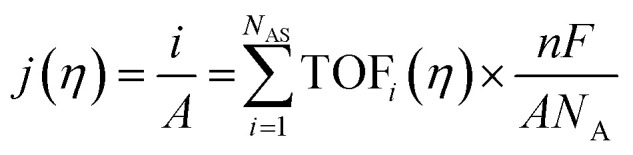
2
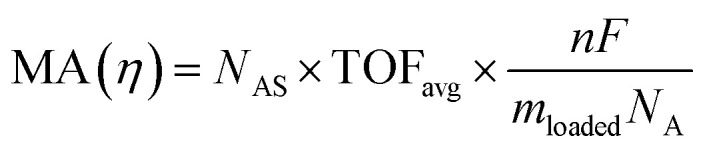
3
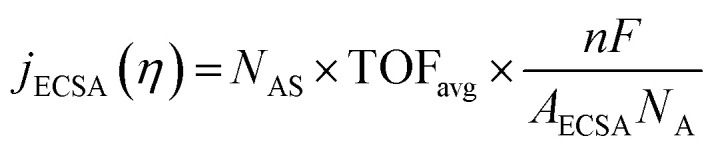
where *j* = total electrode/geometric current density (A cm^−2^), TOF_*i*_ = TOF for active site *i* (s^−1^), *η* = overpotential (V), *F* = Faraday constant (C mol^−1^), *N*_A_ = Avogadro constant (mol^−1^), *A* = geometric area of the electrode (cm^2^), MA = mass activity (A mg^−1^), *m*_loaded_ = loaded mass of the catalyst (mg), *N*_AS_ = number of active sites of the catalyst, *n* = number of electrons transferred per molecule of the product, TOF_avg_ = average TOF for all active sites (s^−1^), *j*_ECSA_ = current density normalized by ECSA (A cm^−2^), and *A*_ECSA_ = electrochemically active surface area (ECSA) (cm^2^).

We note that, particularly in cases where the electrocatalyst has active sites with largely different TOFs, the assumption that all the catalytic sites are equally active can give rise to significant errors associated with per-site TOF estimations. These cases necessitate the explicit estimation of the number of active sites, since specific active sites, although present in minority, can dominate the overall activity of the reaction.^10^ A few approaches have been used by our community for the explicit determination of the “true” number of active sites, including (1) electrochemical oxidation that yields distinct electrochemical features corresponding to the active edge sites and the inert basal plane sites for MoS_2_ catalysts for the HER,^11^ (2) the integration of the area below the redox peak for a redox reaction (M^(*n*+1)+^/M^*n*+^) just before the onset of the OER to estimate the concentration of active sites (M^*n*+^) for 3d transition metal based electrocatalysts,^12^ and (3) the use of surface probes like Pb, Cu, CO and CN that selectively adsorb on certain types of active sites (*e.g.* undercoordinated sites) that have been used to quantify active site densities for single atom catalysts for the ORR,^13^ WS_2_ nanosheets for the HER^14^ and Au catalysts for the CO_2_RR.^15^

**Fig. 1:**
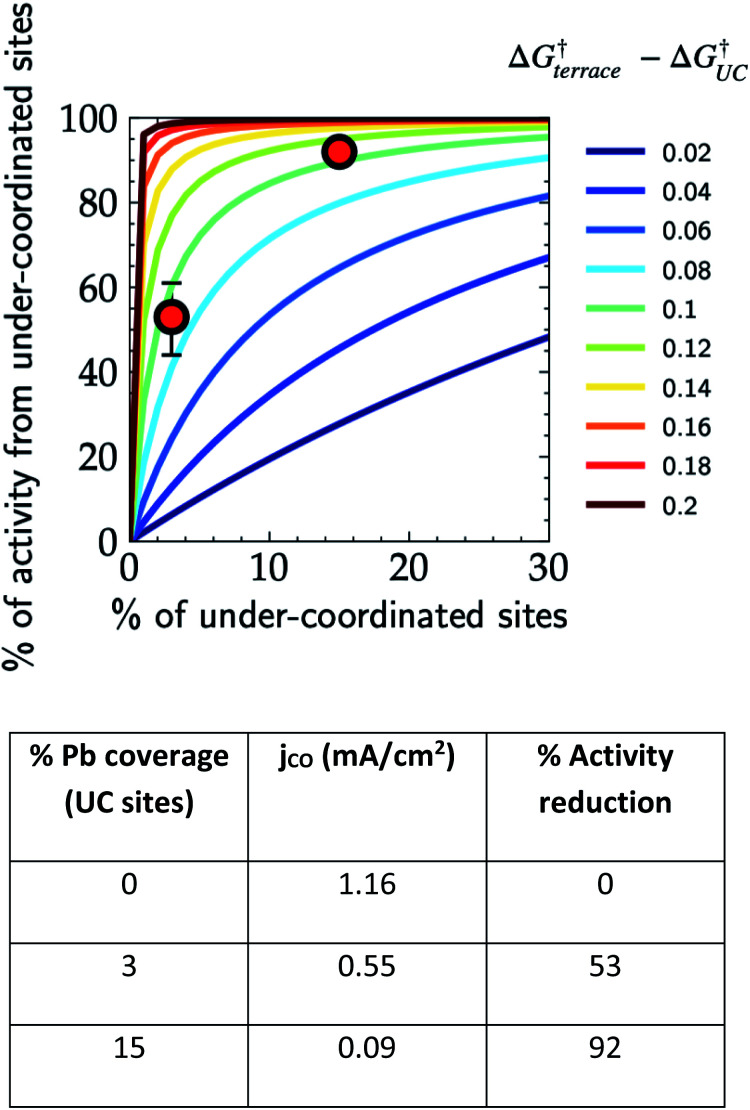
Contribution of under-coordinated sites to the overall activity as a function of their abundance and difference in the activation energy compared to terrace sites. The overlaid red markers correspond to the data shown in the table obtained from Pb UPD experiments on Au(111) single crystals for the CO_2_RR at −0.6 V *vs.* RHE.^15^

In order to demonstrate the importance of explicit estimation of active sites to evaluate and compare the intrinsic activity of electrocatalysts, we turn to the electrochemical CO_2_RR on Au electrodes. Au is one of the most active and selective catalysts for the electroreduction of CO_2_ to CO. Several strategies including alloying, nano/meso-structuring, and ligand grafting have been explored to tune its electrocatalytic activity and selectivity towards CO production. To determine the nature of active sites on Au, Mezzavilla and co-workers performed CO_2_RR activity measurements in combination with Pb-UPD experiments on Au single-crystals.^15^Fig. 1 (bottom panel) shows the observed current densities for CO production (*j*_CO_) at −0.6 V RHE as a function of Pb coverage for Au(111). We use the Pb coverage and *j*_CO_ to calculate the % reduction in activity as a function of reduction in under-coordinated (UC) site density for these surfaces. We overlaid these points onto Fig. 1 (top panel), which shows contours for the contribution towards *j*_CO_ from under-coordinated sites (such as those in Au(110) or steps of Au(211)) *vs.* their relative site density, as determined by the following equation:^16^4
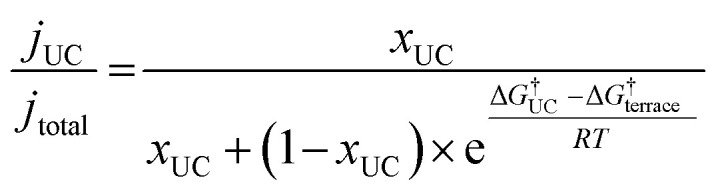
where *j*_UC_ is the current density contribution from under-coordinated sites, *j*_total_ is the total current density, *x*_UC_ is the share of under-coordinated sites (in %), and Δ*G*^†^_terrace_ − Δ*G*^†^_UC_ is the difference in the activation energy of the rate-limiting step between the terrace and under-coordinated sites. We have assumed here that the catalyst surface can be approximated to have these two types of sites.

Considering the intersection of the points from the Pb UPD experiments with the various contour lines in Fig. 1 (top panel), the difference in the barriers between the abundant terrace sites and more active UC sites is between 80 and 120 meV (the range here estimated from the uncertainties in the experimental measurements). Using the Arrhenius law, this energy difference would translate to a 20–100 fold (1–2 orders of magnitude) larger activity of under-coordinated sites compared to terrace sites. Such activity differences are sufficient for the under-coordinated sites to dominate the overall CO_2_R activity on Au.

Another example highlighting the aspect of minority sites dominating the activity was demonstrated for the OER on cobalt oxides.^17^ Using time-resolved FTIR spectroscopy, Frei and co-workers found that the minority defect sites account for most of the observed activity, because the TOF they determined was two orders of magnitude higher than that of a Co surface site assuming all Co sites are active. Using microkinetic modeling, Plaisance and co-workers^18^ found that the active site with the highest TOF changes with the applied potential and at potentials close to the experiments, the minority (311) defect site largely dominates the observed activity in agreement with Frei *et al.* From a modeling standpoint, these examples also highlight the importance of accounting for different active sites, an aspect often overlooked in catalyst screening studies.^10^

In addition to the difficulty in active site estimations, another factor that complicates intrinsic activity measurements and kinetic analysis is the slow mass transport of the reactant/product species to/from the electrode. Since reactant molecules like H_2_, O_2_ and CO_2_ have very low aqueous solubility, the corresponding reactions – HOR, ORR and CO_2_RR are strongly susceptible to mass transport limitations. An additional issue in the case of CO_2_RR is the self-consumption of CO_2_*via* the homogenous reaction with OH^−^ ions to form bicarbonate, which leads to further depletion of reactant concentration.^19,20^ For the HER/HOR on Pt electrodes under acidic conditions, the activity is solely determined by the diffusion of H_2_ even with high rotation rates, owing to the facile kinetics on Pt.^21^ For the ORR on Pt, it has been shown that transport can also affect product selectivity; on low loading Pt/GC electrodes, increasing the electrolyte flow rates resulted in improved H_2_O_2_ selectivity.^22^

These aforementioned examples show that the deconvolution of mass transport effects is needed to obtain meaningful kinetic information. An increasing number of recent studies have employed cell architectures with improved mass transport including floating disc electrode setups,^23^ gas diffusion electrodes (GDE),^24^ membrane-electrode assemblies^25^ and flow cells.^26,27^

**Fig. 2:**
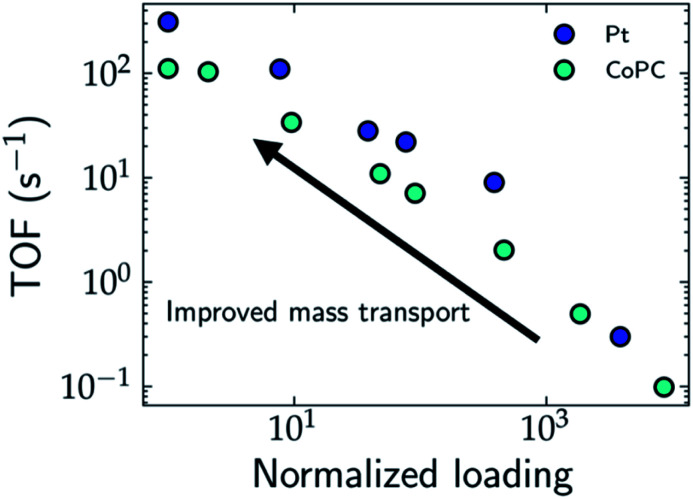
Estimated TOF for the HER and CO_2_RR on Pt (blue) and CoPc (cyan) electrodes, respectively as a function of the normalized catalyst loading. Decreasing the catalyst loading can lead to improved mass transport and limit due to mass transport limitations even at these low loadings. While the TOF of CoPc reaches an upper limit at low loadings, the TOF for Pt does not reach an obvious upper limit due to mass transport limitations even at these low loadings.

One strategy to evaluate the role of mass transport in the apparent activity is to perform measurements by varying the loading of the catalyst. In the limit of ultra-low loading, the effects of mass transport and catalyst segregation are minimized, which results in orders of magnitude increase in the TOF, as demonstrated for Pt nanoparticles for the HER,^28^ single atom cobalt pthalocyanine (CoPc) catalysts for the CO_2_RR^29^ (*cf.*Fig. 2), and Ni/Fe layered oxides and sulphides for the OER, where Chatti *et al.* varied catalyst loading to estimate the intrinsic activity.^30^ In the case of Pt nanoparticles, Hansen and co-workers found that the HER is still mass transport limited at ultra-low loadings, which demonstrates that the frequently reported Tafel slope of 30 mV dec^−1^ results only from H_2_ diffusion without any influence from the intrinsic HER kinetics.^28,31^ For CoPc, the Tafel slope for the CO_2_RR decreases from 230 mV dec^−1^ at high loadings to 120 mV dec^−1^ at low loadings, which highlights the importance of alleviating transport limitations for unambiguous mechanistic conclusions on the rate-limiting steps.

Given the importance of active site estimations in comparing the intrinsic activity of electrocatalysts and in the development of structure–property relationships that are crucial for the design of next generation electrocatalysts, we anticipate that future research efforts are directed towards developing methods to identify and determine per-site TOFs of active sites under fast mass transport conditions. As we recognize that the development of such methods to identify various active sites on the surface is highly challenging, we stress the importance of reporting ECSA/mass normalized activities. They will remain the key metrics to estimate electrocatalytic activity until new easily accessible techniques for the precise quantification of the number of active sites on a broad range of catalysts become available to the community.

While the main focus of this perspective is on electrocatalytic activity, long term stability of an electrode material is also a crucial performance metric for practical applications. Methods to determine long term stability are only beginning to receive attention,^32^ with metrics like the *S*-number proposed for the OER recently^33^ that are also applicable to other electrocatalytic transformations.^34^ The *S*-number is defined as the ratio between the amount of product generated and the amount of dissolved active sites. The *S*-number is akin to the well-known turnover number that is widely used by the homogeneous and heterogeneous catalysis communities. Given the importance of stability as a crucial performance metric of an electrocatalytic system, future research in understanding degradation pathways and rigorous stability testing is needed for industrial application.

## ECSA normalized activities and TOFs suggest small changes in intrinsic catalytic activity so far

**Fig. 3:**
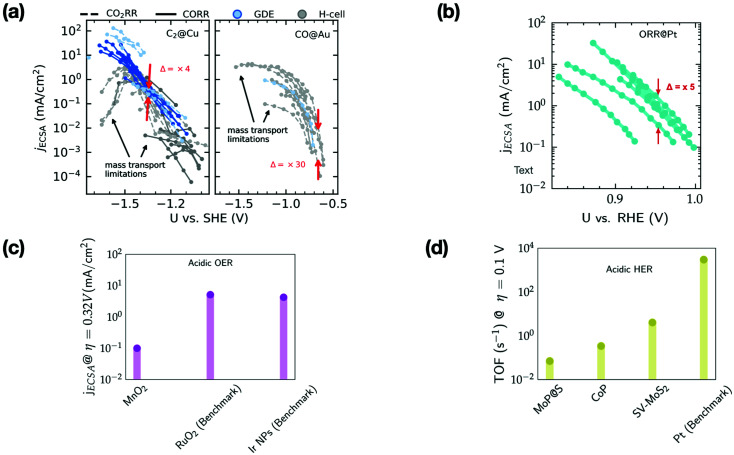
(a) ECSA normalized current density (*j*_ECSA_) for CO_2_ and CO reduction (CO_2_R) to C_2_ products and CO_2_R to CO on various nanostructured/modified Cu^35–50^ and Au electrodes,^51–59^ respectively. The data include experiments performed in GDEs and H-cells, (b) ECSA normalized current density for the ORR on nanostructured/Pt alloys.^60–66^ Δ represents the variation in ECSA normalized activity in the kinetically controlled regions. Regions limited by mass transport are highlighted by arrows. (c) ECSA normalized current density at an overpotential (*η*) of 0.32 V for MnO_2_ electrodes (based on earth-abundant Mn) compared to the benchmark Ir/Ru based electrodes for the OER in acid^67–69^ (d) TOF at an overpotential (*η*) of 0.1 V for the HER in acid on several non-precious metal electrodes compared to the benchmark Pt.^28,70–72^

Fig. 3 shows the ECSA-normalized activities reported for several nanostructured/modified Cu,^35–50^ Au^51–59^ and Pt^60–66^ electrodes that are state-of-the-art electrocatalysts for the CO_2_RR (panel a) and ORR (panel b), as well as those for OER electrocatalysts^67–69^ (panel c), and the TOFs for HER electrocatalysts (panel d).^28,70–72^ Note that the benchmark catalysts for these transformations (with the exception of Cu) are typically based on precious metals like Au, Pt, Ir and Ru. For the CO_2_RR and ORR, the kinetically controlled data (low overpotential region) in Fig. 3a and b show only small differences in the ECSA-normalized activity among catalysts of various morphology or modification (Δ = 5–30).

Based on the small variations in ECSA normalized activity, we suggest that these nanostructuring/electrode modification strategies do not alter the intrinsic activity of these materials, as has been noted in previous studies.^37,73–75^ These strategies increase the active site density resulting in higher geometric current densities compared to their planar counterparts. However, these electrodes quickly reach mass transport limitations (*cf.*Fig. 3a and b), which places an upper bound on the geometric current density that can be achieved.

The ECSA normalized activity and TOFs of earth abundant catalysts for the OER and HER under acidic conditions (*cf.*Fig. 3c and d) are *ca.* 2–3 orders of magnitude *less* active than benchmark electrodes based on precious metals. The reduced cost of these earth abundant catalysts compared to precious metals would render increased catalyst loadings viable for commercial application. However, in many cases this would still impede overall performance since charge/mass transport limitations in very thick catalyst layers could restrict the achievable geometric current densities. An effectively reduced geometric activity would then result in higher operational cost compared to precious metal based electrolyzers.^7,76^ Therefore, the intrinsic activity of these earth abundant catalysts has to be significantly improved before they can be practically relevant for the scale-up of electrolyzers and photoelectrochemical devices.^7^

Despite decades of research, the development of electrocatalysts for these important transformations has so far achieved only incremental improvements in intrinsic activity (and overpotentials).^1^ These observations emphasize the need to develop next generation electrode materials with significantly higher intrinsic activities than the benchmark catalysts that are typically based on precious metals. Previous studies have suggested that moving beyond traditional electrode materials (*i.e.* metals/metal oxides) including single atom catalysts,^77^ high entropy alloys,^78^ graphite-conjugated catalysts,^79^ and MXenes^80^ opens new avenues for electrode design. Additionally, strategies including the introduction of a third dimension (normal to the surface *e.g.* ligand scaffolds, confinement, *etc.* that can interact geometrically/chemically with the reaction intermediates),^81^ the use of external forces^82^ and operation under dynamic potentials^83^ have also been suggested to be promising directions in this regard. TOF evaluations will be ever important for rigorous comparison of intrinsic activities in all these cases and move the field forward in the right direction towards new materials and design strategies.

## Arrhenius law: small steps mean giant leaps, and what it means for DFT simulations

Having established the fact that, in terms of ECSA normalized activity/TOFs, the variations in the activity of new *vs.* benchmark catalysts for the CO_2_RR/ORR/OER/HER vary within 1–2 orders of magnitude, we discuss the capacity of computational models to predict differences in intrinsic activity. Using the Arrhenius law (eqn (5))5
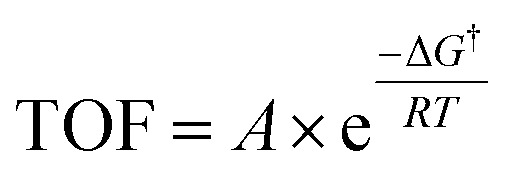
where *A* is the prefactor, Δ*G*^†^ is the activation barrier, *R* is the universal gas constant and *T* is the temperature, we find that a shift in 1–2 orders of magnitude in TOF translates to a decrease in the activation barrier of the rate limiting step by ∼0.1–0.15 eV at 300 K compared to the benchmark catalysts for these reactions (*cf.*eqn (6)). Therefore, as stated earlier, the small changes in ECSA normalized activity seen for CO_2_RR and ORR catalysts obtained by nanostructuring/modifications (*cf.*Fig. 3a and b) likely arise from differences in the active site densities and not from active sites with intrinsically different activity. We note that the simplified expression for TOF used in eqn (5) is only meant to demonstrate the general sensitivity of TOFs to the reaction energetics. Ideally, detailed microkinetic models including the consideration of adsorbate coverage effects and different reaction pathways for a given electrocatalyst material are needed to gain mechanistic insights, as has been highlighted in recent studies.^84–86^6
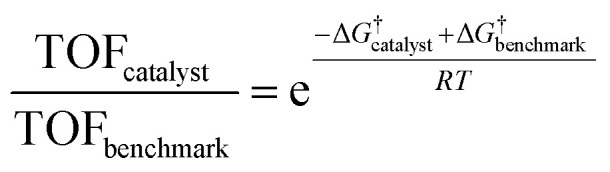


The typical errors associated with estimating gas-phase reaction energetics using density functional theory (DFT) simulations involving approximate exchange-correlation (XC) functionals are usually around 0.15 eV.^87^ The errors associated with the description of certain types of gas-phase molecules and adsorbates can be larger, and correction schemes have be proposed in this regard.^88,89^ Note that 0.15 eV already translates to an error of 300× in the TOFs using eqn (5). This uncertainty in TOF is already higher than the 1–2 orders of magnitude differences in ECSA-normalized activity as noted in the previous section. In modeling electrocatalytic reactions, these errors are further compounded by the electrochemical nature of the reactions and the complex electrode–electrolyte interface.^90^ As a result, a number of approximations are required in computational electrochemistry including (i) the absolute potential of the standard hydrogen electrode,^91^ (ii) the inclusion of the effects of the aqueous solvent and ions on the reaction energetics using mixed implicit/explicit approaches,^92^ and (iii) the need for a grand-canonical framework for the estimation of electrochemical barriers at constant potential that is still under active development.^93–95^ While these approximations underscore the difficulty in quantitative predictions of electrocatalytic activity using DFT simulations, theory might predict relative differences in activation barriers within a given reaction mechanism and capture trends in activity for several catalysts with much less uncertainty due to error cancellation.^87,96^ This fortuitous error cancellation is likely the reason for the success of electrocatalyst screening studies in rationalizing and predicting promising candidates for a number of reactions.^1^ In many instances, these predictions have also been validated by experiments.^72,97–100^

**Fig. 4:**
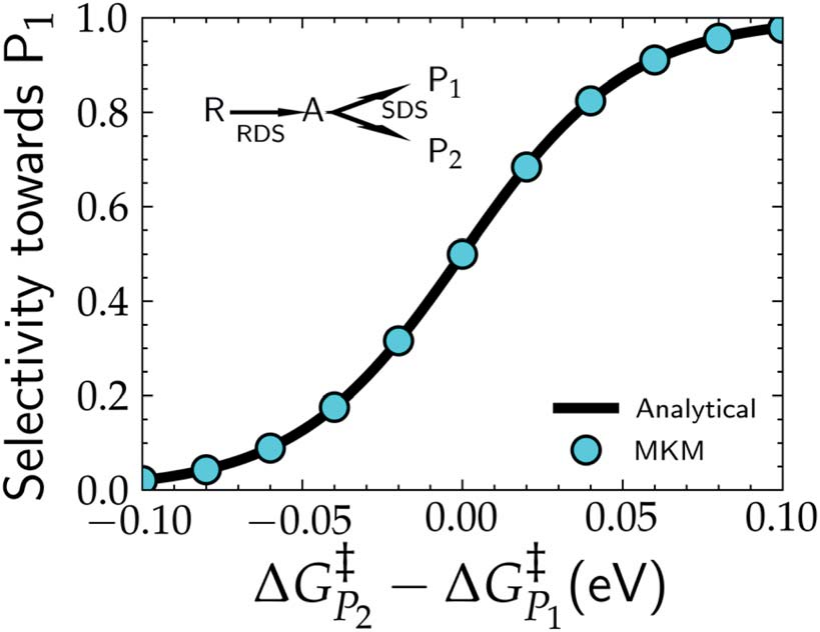
Selectivity predictions in a model reaction network consisting of a rate determining step (RDS) followed by a selectivity determining step (SDS) leading to the two products, P_1_ and P_2_. Cyan points show the results in the selectivity towards P_1_ (*S*_P_1__) resulting from a microkinetic model (MKM), with *S*_P_1__ calculated *via*eqn (8), while the solid black line represents the same selectivity calculated from the analytical eqn (9).

In addition to theoretical activity predictions, numerous attempts have been made to explain changes in selectivity towards different products *via* computational models.^101–104^ Similar to the issue in estimating the activity of catalysts, product selectivity is strongly sensitive to the calculated free energies.^105^ This is illustrated in Fig. 4, which shows the results obtained for selectivity estimations towards the products P_1_ and P_2_ for the model reaction network shown in the inset, as a function of the difference between their activation free energies, 
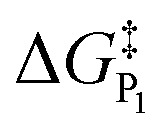
 and 
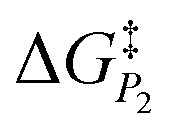
. We show the results from a toy microkinetic model (MKM, *cf.* Appendix for details) and an analytical model.

The selectivity towards P_*i*_ (*S*_P_*i*__) has been calculated from the MKM model as:7
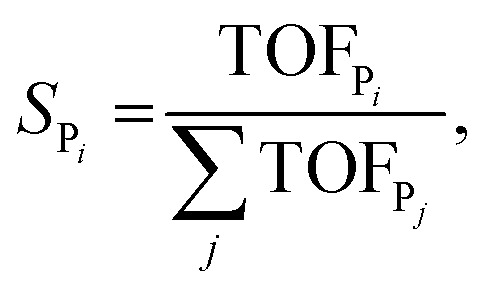
where TOF_P_*i*__ is the turnover frequency towards product *i*, and the denominator is the sum over all the possible products *j* from the reaction. For the two-product model used in this example, this reduces to8
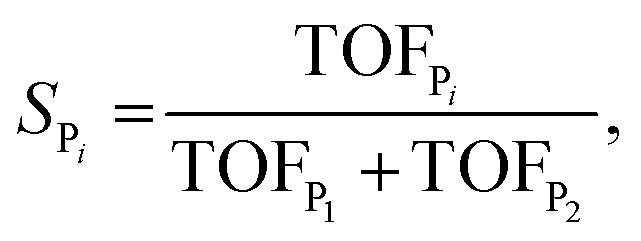


The selectivity can also be determined analytically (solid lines in Fig. 4) as:9
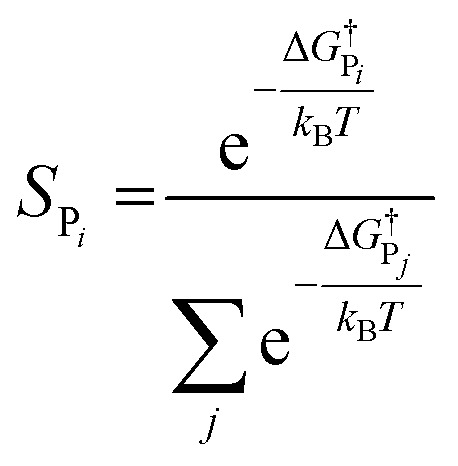


Note that in eqn (9) we only include the activation free energies involved in the selectivity determining step (SDS), while eqn (8) contains the computed TOFs from the MKM, which includes the entire reaction pathway. Both models lead to an identical result as can be seen in Fig. 4.

We find that the difference in the activation energy (Δ*G*^†^_P_) between the two products P_1_ and P_2_ needs to be well within 0.1 eV in order for both products to be produced with non-negligible selectivity. In fact, in the region from −0.05 eV to 0.05 eV, the selectivity towards P_1_ already changes from 12% to 87%. Therefore, selectivity changes towards different products that are observed in experiments (*e.g.* CO_2_RR towards multi-carbon products) give rise to magnitudes of changes in reaction energetics so small they are well below typical errors in DFT simulations. Similarly, the strong sensitivity of the selectivity to the activation energy changes suggests that it is highly unlikely that small increases in selectivity (10–20%) arise from changes in the active sites of the catalyst, which would give rise to large changes in the reaction thermodynamics/activation barriers. We suggest that it is therefore virtually impossible for DFT simulations to predict product selectivities with reasonable uncertainties unless one product dominates by >99%. Additionally, any theoretical predictions of selectivity determining steps towards various products with corresponding differences in free energy beyond 0.05 eV would imply the complete predominance of the activity towards one product (*i.e. S*_P_*i*__ > 87%).

In electrochemical reactions, product selectivity can also be determined from the competition between two chemical steps (C–C) or between a chemical and an electrochemical step (C–EC). While the selectivity will be potential-independent in the case of C–C, the applied potential can have a strong impact on selectivity in the case of C–EC. An example in this regard is the selectivity of acetate *vs.* other C_2_ products on Cu as discussed in a recent study by Heenen and co-workers.^106^ Two SDS were included in the reaction mechanism, one that involved a solution phase reaction and the re-adsorption of a ketene species (C–C), and the other involving a PCET step towards other C_2_ products *vs.* the re-adsorption of the ketene species. The different potential dependence of these SDS is crucial to explain the complex U-shaped potential dependence of acetate selectivity observed in experiments.^45^ It is therefore imperative that computational studies use microkinetic modelling to consider the implications of the competition between chemical and electrochemical steps while proposing SDS and branching pathways in the reaction mechanism.

In summary, computational models are powerful tools in providing atomistic insights on the active sites, understanding reaction mechanisms and predicting trends in electrocatalytic activity, thereby providing clear guidelines for rational catalyst design. However, we add a note of caution that must be kept in mind: the uncertainties in the computed TOFs span orders of magnitude, due to (1) the errors present in XC functionals applied in surface science computations, which are compounded by (2) the several approximations often used in models of the electrochemical interfaces, and (3) the sensitivity of the TOF to the energetics owing to the Arrhenius law. Additionally, the error bounds in computed energetics also preclude a precise determination of product selectivities. Moving forward, we should complement simulations with sensitivity analyses and uncertainty estimations while performing mechanistic/screening studies. It is encouraging to note that a number of recent studies have taken a step in this direction.^96,107,108^ Further, we anticipate that multi-scale modeling studies that combine *ab initio* kinetics with continuum transport models that are evaluated against experiments performed under well-defined conditions will become increasingly important in obtaining a thorough understanding of electrochemical reaction mechanisms.^51,106^

## Electrolyte engineering: complexity offers opportunities for electrocatalyst design

**Fig. 5:**
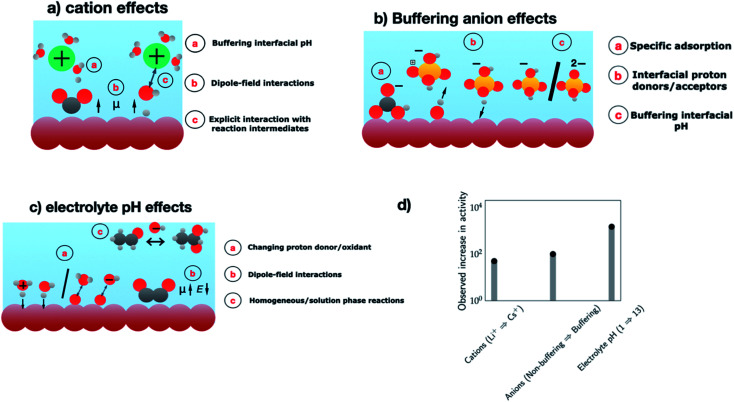
Schematics showing the different sources of electrolyte effects involving several aspects of the electrolyte environment. (a) Cation effects, (b) anion effects, and (c) electrolyte pH effects. Colour coding – electrode (brown), electrolyte (blue), hydrogen (white), oxygen (red), carbon (grey), phosphorous (yellow) and cations (green). (d) Observed increase in the activity for each of the electrolyte components – CO_2_RR to C_2_ products on Cu electrodes at −1 V *vs.* RHE (cations, Li^+^ → Cs^+^),^112^ CO_2_RR to CH_4_ at −0.8 V *vs.* RHE (non-buffering → buffering anions),^116^ and ORR on Au(100) at 0.8 V *vs.* RHE (pH 1 → pH 13).^126^

In the past two decades, the majority of the research efforts have been directed towards electrode design, while the role of the electrolyte in improving catalytic activity and tuning product selectivity has only been explored more recently. As the electrocatalytic system consists of the electrode and the electrolyte, an overall understanding of the system also requires the consideration of the electrolyte environment. Such an understanding can aid in the complete optimization of the electrocatalytic system for the processes of interest. Several aspects of the electrolyte environment: cation identity, buffering anions, and electrolyte have all been demonstrated to affect the catalytic activity and product selectivity for a number of electrochemical transformations. To set the stage for the discussion, Fig. 5 summarizes the different sources of electrolyte effects and the resulting activity enhancements observed for several electrochemical transformations. In the following, we highlight a few examples of these effects.

### Effects of cations

Several studies have reported a significant impact of the cation identity on catalytic activity and/or product selectivity for electrochemical transformations including the HER, OER, ORR and CO_2_RR. Recent studies have shown 5–10 fold enhancements with cation identity in alkaline HER activity on several metal electrodes.^109^ For Pt and Ir electrodes, HER activity was found to decrease from Li^+^ to Cs^+^, while on Au, Cu and Ag electrodes the opposite behavior has been reported. The exact reason for this behavior is still unclear. A similar 10-fold increase in oxygen evolution activity with increasing cation size was observed on nickel oxyhydroxides – NiOOH (Li^+^ to Cs^+^)^110^ and oxygen reduction activity on Pt/C and LaMnO_3+*δ*_ (Li^+^ to K^+^).^111^ CO_(2)_R has also been shown to be particularly sensitive to cation identity, where up to a 15–50 fold increase in activity from Li^+^ to Cs^+^ has been reported for CO_2_ reduction to CO on Ag and CO reduction to C_2_ products on Cu,^112^ while the rate of methane production and HER is essentially unaffected by cation identity.^113^ This also highlights the ability of cations to tune the selectivity towards specific products.

Several hypotheses have been used to rationalize these cation effects, including cation blocking of the active sites, chemical interactions between the cations and the relevant reaction intermediates, dipole-field interactions with the adsorbed species, cations altering the interfacial water structure and cations buffering the interfacial pH (*cf.*Fig. 5a, cation effects).^114^ While the exact reason for the observed cation effects will depend on the reaction studied where a combination of these hypotheses could be at play, previous work by Ringe and co-workers has shown that the observed trends in cation effects for a diverse set of experimental data including CO_2_RR can be explained by the dipole-field model.^112^ According to this model, cations with a small hydrated radius like Cs^+^ have higher concentrations near the electrode due to lesser repulsive interactions compared to a cation with a larger hydrated radius like Li^+^, which results in a higher surface charge density and stronger interfacial field that stabilizes the decisive reaction intermediates like *CO_2_ and *OCCO, thereby resulting in increasing CO_2_RR activity with decreasing hydration radius. Note that such activity enhancements (*ca.* 1–1.5 orders) observed in changing cation identity (*cf.*Fig. 5d) are on par with those seen in Fig. 3c and d, which compares electrode materials with benchmark catalysts and highlights the opportunities offered by cations in modulating electrocatalytic activity and tuning product selectivity.

### Effects of buffering anions

Buffers are usually used to regulate the interfacial/solution pH of an electrocatalytic system to a desired value. However, buffering anions can also affect the electrocatalytic activity *via* direct involvement in the reaction. For instance, buffers can act as proton donors in reduction reactions, proton acceptors and oxygen donors in oxidation reactions, or poison the catalyst surface *via* specific adsorption (*cf.*Fig. 5b, buffering anion effects).

In several cases, buffers can outcompete H_2_O as the proton donor, which results in activity improvements for reduction reactions under neutral/alkaline conditions that are limited by proton–electron transfer. Jackson and co-workers investigated the reaction orders of phosphate (H_2_PO_4_^−^) and borate (B(OH)_3_^−^) buffers for the HER on polycrystalline Au electrodes.^115^ They found that phosphate anions, unlike borate, outcompete H_2_O as a proton donor under near neutral pH conditions (6–8.5), thereby accelerating the rate of HER by approximately 5–10 fold at *ca.* −0.4 V *vs.* RHE. The strong promotional effect of phosphate is further highlighted by the ability of a 2.8 M phosphate buffer at neutral pH to rival the HER activity under acidic conditions (pH 1). This is an exciting finding since (i) the kinetics of the HER are typically sluggish under neutral/alkaline conditions, where H_2_O is the proton donor, and (ii) it demonstrates that buffers can be used to promote HER kinetics in neutral environments for electrodes that are not stable under acidic conditions, without compromising on the activity.

Resasco and co-workers reported similar observations of anion effects for CO_2_ reduction on Cu electrodes.^116^ They found the composition and concentration of buffering anions have a significant effect on H_2_ and CH_4_ production but little/no effect on CO, HCOO^−^, C_2_H_4_ and C_2_H_5_OH. This finding is consistent with the fact that the former products are likely limited by proton transfer while the latter are not. They furthermore proposed that the buffering anions can directly participate in the reactions as proton donors, since the ability of buffers to modulate the interfacial pH alone was insufficient to explain the observed differences in activity towards H_2_ and CH_4_. This effect results in a *ca.* two orders of magnitude increase in the current density towards CH_4_ at −0.8 V *vs.* RHE for electrolytes with buffering *vs.* non-buffering anions.

Buffers can also participate in electro-oxidation reactions as proton acceptors, as demonstrated by Surendranath and co-workers for the OER on Co based catalysts.^117^ The OER activity and the potential dependence (measured by Tafel slopes) were enhanced in the presence of a phosphate buffer. In this case, they showed that phosphate outcompetes H_2_O and OH^−^ as a viable proton acceptor under near neutral pH conditions.

More recently, Marcandalli *et al.* proposed that carbonate buffer (CO_3_^2−^) can act as an indirect oxygen donor during CO oxidation on Au electrodes, by generating OH^−^ species *via* acid/base equilibrium reactions.^118^ They observed a reduction in the overpotential for CO oxidation by *ca.* 0.3 V in the presence of carbonate buffer, compared to the situation where H_2_O alone is the oxidant.

In addition to accelerating interfacial reduction/oxidation reactions, buffers can also have a negative effect on catalytic activity *via* specific adsorption on the electrode surface. For instance, Zeng and co-workers demonstrated a dual role of the bicarbonate (HCO_3_) buffer in the electrochemical CO_2_RR, catalyzed by cobalt phthalocyanine (CoPc) electrodes.^119^ They suggest that HCO_3_^−^ poisons the active sites *via* electrosorption at low overpotentials, and accelerates the CO_2_RR towards CO at high overpotentials by participating as a proton donor in the rate-limiting step. A similar inhibitory role of phosphate buffer in the partial current density towards CO during the CO_2_RR on Au electrodes was also observed by Wuttig and co-workers,^55^ and during ethanol oxidation on Au electrodes by Lai and co-workers.^120^ These inhibitory effects are likely related to the specific adsorption of phosphate anions on Au electrodes, thereby blocking active sites for these reactions.

In summary, buffering anions can strongly alter the activity of several electrocatalytic reactions *via* their direct involvement as proton/oxygen donors, proton acceptors or as poisons. Fig. 5d shows that the observed activity enhancements can, in some cases, be up to two orders of magnitude. These observations highlight that a judicious choice of the buffering anion that includes consideration of optimal pH and mass transport conditions is needed in order to maximize the flux of the buffering anions at the interface,^121^ which can improve electrocatalytic activity and tune the product selectivity of electroreduction/oxidation reactions. While previous studies have highlighted stability issues associated with buffered electrolytes for the large-scale deployment of electrolyzers,^122^ we anticipate that the exciting opportunities offered by buffering anions can motivate future studies in developing high current density electrode configurations suitable for the use of buffered electrolytes.

### Effects of the electrolyte pH

Electrolyte pH has shown to have a significant effect on the electrocatalytic activity and product selectivity for a number of electrocatalytic reactions. These pH effects mainly manifest in three possible ways: (1) change in the proton donor/oxidant, (2) homogeneous/solution phase reactions catalyzed by OH^−^ ions, and (3) dipole-field interactions, as indicated in Fig. 5c, “electrolyte pH effects”.

A change in the electrolyte pH can result in a change in the proton donor/oxidant for reduction/oxidation reactions. For instance, for the HER on Pt, Au and Ir electrodes in unbuffered electrolytes, the activity decreases with an increase in the electrolyte pH.^123^ This effect was suggested to arise from a change in the proton donor from H_3_O^+^ ions under acidic conditions to H_2_O for pH > 3 as the H_3_O^+^ ions quickly reach diffusion limitations.^124^ The proton donor activity correlates with the activation barrier for the Volmer/Heyrovsky reactions involving an O–H bond cleavage. The activation barrier for these reactions is therefore higher for H_2_O compared to H_3_O^+^ ions (p*K*_a_(H_2_O) ≫ p*K*_a_(H_3_O^+^)), which yields sluggish HER kinetics under conditions where H_2_O is the dominant proton donor.

Another way the electrolyte pH can alter activity is *via* dipole-field interactions. At a fixed potential *U vs.* the reversible hydrogen electrode (RHE), the absolute potential (*U vs. e.g.* SHE) shifts by −59 mV per pH. The interfacial field is proportional to the absolute potential, and therefore it shifts with the electrolyte pH at a given *U vs.* RHE. Adsorbates with large dipole moments and/or polarizabilities can strongly interact with the interfacial field, thereby resulting in sizeable changes in their adsorption/transition state energies. An important example in this regard is the CO_2_RR to C_2_, which is limited by the initial CO dimerization step.^113^ This step is stabilized by the interfacial field and the stabilization increases with decreased absolute potential. The dependence of the activity on absolute potential results, on an RHE scale, in an overpotential of *ca.* 0.36 V towards C_2+_ products under alkaline conditions (pH 13) compared to neutral conditions (pH 7).^125^ At −0.6 V *vs.* RHE, there is over three orders of magnitude increase in C_2_ activity between these two pH values.

Remarkable pH effects have also been observed for ORR activity on Au(100) electrodes by Markovic and co-workers.^126^ The authors reported up to four orders of magnitude increase in the ORR current density at a given *U vs.* RHE under alkaline conditions (0.1 M KOH, pH 13) compared to acidic conditions (0.1 M HClO_4_, pH 1). Modeling studies attribute this pH effect to the strong field stabilization of the *OOH species involved in the ORR.^127,128^

Finally, the electrolyte pH can also affect activity by triggering homogeneous/solution phase reaction pathways without the direct involvement of the electrocatalyst. These reactions are usually catalyzed by OH^−^ species under strongly alkaline conditions. Examples include:

(i) The electro-oxidation reactions of a number of alcohols on Au electrodes, which are key reactions in direct alcohol fuel cells. These reactions show a clear correlation between the p*K*_a_ of the alcohol and the onset potential for alcohol electro-oxidation on Au electrodes.^129^ This correlation was attributed to the OH^−^ catalyzed proton transfer of the alcohol (R–OH) to form the reactive alkoxide species (R–O^−^), which undergoes further oxidation at the electrode. As a result of the OH^−^ catalyzed solution phase reaction, a 0.36 V reduction in the overpotential for ethanol electro-oxidation and a *ca.* 10 fold increase in peak current density were observed by Lai and co-workers under alkaline conditions compared to acidic conditions.^120^

(ii) The Cannizzaro disproportionation reaction, which involves involving the reaction of aldehydes with OH^−^ ions to form alcohols and acids. An example here is the production of formic acid and methanol during the CO_2_R on boron doped diamond electrodes that are generally inactive for this reaction.^130^

(iii) The solution phase reaction of a ketene species (CH_2_CO) with OH^−^ ions during the CO_2_RR on Cu electrodes that is proposed as the major pathway towards the formation of acetate.^106^

In all three cases, the solution phase reactions are catalyzed by the concentration of the OH^−^ ions present in highly alkaline environments. The rate constants corresponding to the pathways involving OH^−^ are usually orders of magnitude higher than those with H_2_O. Importantly, the nature of the electrocatalyst does not play a direct role in these reactions.

The highlighted examples of the observed activity enhancements due to the involvement of the electrolyte environment are on par/larger than those achieved with electrode modifications so far, and clearly indicate the exciting opportunities offered by electrolyte engineering. Therefore, we strongly advocate for research efforts directed towards further understanding the complex electrode–electrolyte interface both using *in situ*/*operando* techniques and computational models that include actively include the various aspects of the electrolyte environment either in an explicit or a mean-field manner.

## Concluding remarks

Identifying and developing electrocatalysts with high intrinsic activity are crucial to accelerating the transition towards the sustainable production of fuels and chemicals. In this perspective, we discussed the importance and challenges associated with intrinsic activity estimations. We also highlight the issues associated with slow mass transport of the reactant and/or product species on accurate intrinsic activity estimations and mechanistic interpretations and discuss possible mitigation strategies in this regard. A thorough analysis of activity estimations reported in the literature points to modest improvements in the intrinsic activity for several electrochemical conversions observed thus far compared to benchmark catalysts for these reactions. These modest changes highlight the need for a paradigm shift in the design and discovery of electrocatalysts.

From a modeling perspective, we discuss the strong sensitivity of the reaction energetics on activity and selectivity estimations, the sources of errors arising from computational methods, and the approximations made in models of the electrode–electrolyte interface. These errors preclude quantitative estimations of activity and selectivity, which emphasizes the importance of complementing models with sensitivity analyses and uncertainty quantification.

In determining product selectivity, the importance of considering the competition between chemical and electrochemical steps is highlighted. Considering the present degree of accuracy in the employed computational models, we suggest that simulations be evaluated with experiments performed under well-defined conditions when possible. Finally, we discuss the exciting opportunities offered by the electrolyte environment by highlighting several examples of activity enhancements involving different electrolyte components including cations, buffering anions and the electrolyte pH. While the largest improvements in intrinsic activity are likely to come from the design and development of next generation electrocatalysts, we anticipate that electrolyte engineering will play an important role in the complete optimization of electrochemical systems, thereby paving the way for widespread penetration of sustainable energy conversion technologies.

## Data availability

All the raw data needed to reproduce Fig. 1 and 4 in the article are available at https://github.com/CatTheoryDTU/chemsci_perspective.

## Author contributions

N. G. and K. C. conceived the idea and formulated the outline of the perspective. All authors contributed to the discussion and writing of the manuscript.

## Conflicts of interest

There are no conflicts to declare.

## Appendix: details of the microkinetic model

The microkinetic model was solved using the CatMap package.^131^ The included reactions in the model were:R +* ↔ A* (RDS); Δ*G*_R_ = 0 eV, Δ*G*^†^_R⃑A*_ = 0.773 eV, Δ*G*_A*_ = 0 eV
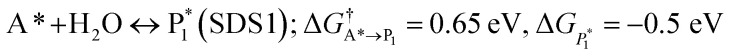

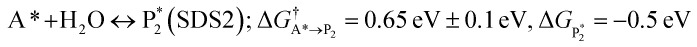

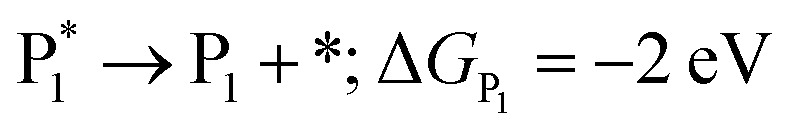

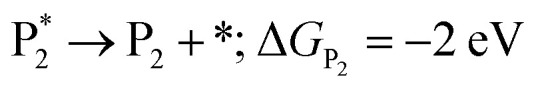
(Δ*G*_H_2_O_ = 0 eV)

The temperature was set to 300 K and the gas phase pressures of *R* were set to 1 bar. An equal pre-factor for the TOFs of 1 × 10^13^ s^−1^ was used for every elementary step.

In order to estimate the selectivity shown in Fig. 4, the reaction barrier towards 
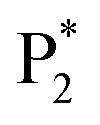
 was varied within ∓0.1 eV and the resulting TOFs towards P_1_ and P_2_ were used in eqn (8).

## Supplementary Material
